# Exploring the Rice Cultivars in Large-Scale Chinese Local Gazetteers: A Computational Approach

**DOI:** 10.3390/plants11233403

**Published:** 2022-12-06

**Authors:** Yuehua Li, Hui Li

**Affiliations:** 1 College of Information Management, Nanjing Agricultural University, Nanjing 210095, China; 2 College of Humanities and Social Development, Nanjing Agricultural University, Nanjing 210095, China

**Keywords:** local gazetteers, rice cultivars, computational approach

## Abstract

Chinese local gazetteers have long been widely used by scholars to investigate the local products, culture, economy, and much more. Confronted with large-scale digitized resources nowadays, researchers can explore historical texts in a novel way. In this paper, we propose a computational approach in order to perform large-scale quantitative analysis of plant knowledge embedded in Chinese local gazetteers. We select the typical rice cultivars by their occurrences in the records, interpret their common features, and leverage the data clustering algorithm to investigate the inner connections among cultivars. We conduct a case study on a dataset of records of rice cultivars over 8 centuries in Jiangsu Province, China. We find that although planting early-season rice in Jiangsu province was the common practice, the local rice farmers cared more about the color, quality, and uses of cultivars than their sowing time. In addition, not all the rice varieties mentioned frequently in records are local plants. Plants imported from other provinces or countries were also highly recorded because of their good quality and special characteristics. Our study offers a practical guide and reference to history study as well as useful clues for modern agriculture.

## 1. Introduction

A local gazetteer (difangzhi) has been one of the major sources for studying Chinese history and provides comprehensive descriptions of a locality in China [1]. For more than two thousand years, gazetteers have been continually written or compiled by local officials and scholars, concerning a variety of topics such as local products, landscape, population, economy, and culture. By studying these records, researchers can not only gain valuable insights into the cultural, economic, and political dynamics in pre-modern China, but also uncover more clues about how different forms of life evolved and how ecosystems changed over time and space.

Assisted by the digitization of historical texts, scholars are increasingly paying attention to the text mining of large-scale local gazetteers nowadays. Most of the previous studies focused on the extraction and organization of entity information in historical texts, such as names and descriptions of persons, plants, and locations. For instance, Chen et al. [2] developed LoGaRT, a suite of tools (e.g., text search and map visualization), for studying Chinese local gazetteers; Liu et al. [3] employed natural language processing techniques to extract person names, location names, and their relationships. However, only a few studies have attempted to further interpret the latent knowledge about products (e.g., plants and animals) in gazetteers.

This paper presents a computational approach for information exploration of plant records in large-scale Chinese local gazetteers, and an on-going study of rice cultivars in Jiangsu Province of China on the basis of a digitized collection in Nanjing Agricultural University (NJAU). Our objective is to extract and exploit the knowledge about the rice cultivars embedded in the records of historical gazetteers. In addition, we are especially interested in answering the following two questions:

- What are the rice cultivars mentioned most frequently in local gazetteers of a certain region in China, e.g., Jiangsu Province? Are there any common features shared by these varieties?
- Among these cultivars, are there any non-local varieties? What characteristics do they have?

## 2. Materials and Methods

Jiangsu province has a long history of being agricultural, economic, and cultural center of southern China since ancient times. With the fertile farmland, abundant lakes, and rivers, Jiangsu is also named as “the land of rice and fish”, and roughly three-fifths of the total arable land are in paddy (wet-rice) fields [4].

### 2.1. Dataset

Since the 1950s, historians and agronomists in NJAU have devoted to archiving and digitizing information related to local products from over 9000 volumes of Chinese historical gazetteers. Currently, the digitized collection in NJAU covers detailed records of rice cultivars in Jiangsu Province spanning from the 12th to 20th century. Each record in this digitized collection mainly consists of a rice name, the local gazetteer where the rice is recorded, the time when the gazetteer was published, and the corresponding rice description.

The data collection originally has over 6000 records of rice cultivars in Jiangsu Province, and one-third of them have only rice names without descriptions. In this study, we only focus on the records with concrete rice descriptions.

### 2.2. Methods

**Figure 1:**
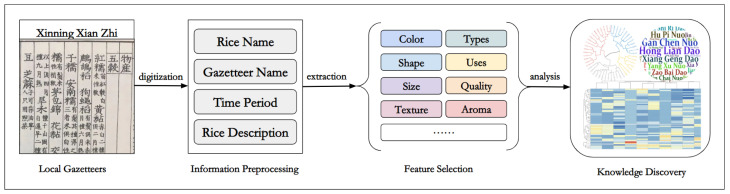
A general overview of our workflow.

The computational approach of our study is comprised of three major steps: information preprocessing, feature selection, and knowledge discovery. Figure 1 shows a general overview of our approach and the details of the major steps are listed as follows.

- **Information Pre-Processing**. Due to dialects, naming conventions, social customs and habits, multiple rice cultivars with different names might refer to one single variety or multiple rice cultivars share the same name. In order to solve this problem, we compare the text similarities between each description and set a threshold to filter the obviously irrelevant ones. For instance, if two descriptions heavily overlap (similarity score over 95%) and the corresponding rice names are mentioned as alternative names for one another, these two cultivars are marked as the same and merged into one record. After experts in this field evaluate the results, the verified records are updated in the dataset. Admittedly, there are a few cultivars that even experts cannot distinguish easily based on descriptions. We currently regard them as different varieties and set separate IDs for each of them. In our experimental dataset, only less than 5% of records in Jiangsu Province remain to be disambiguated, which we think will not have a huge impact on our current study.
- **Feature Selection**. A rice description usually contains feature information such as appearances (e.g., shell/rice color, shape and size), growth duration (early/mid/late season), type (japonica/glutinuous/indica), texture, aroma, quality, etc. Experts also participate in this task to help us set ten dimensions which correspond to different features embedded in the rice descriptions. They manually annotate the rice features according to each record description and assign them to different dimensions, respectively.
- **Knowledge Discovery**. In order to discover the latent knowledge embedded in rice records, we employ machine learning techniques such as clustering to the dataset. The goal of cluster analysis is to discover the groups of data by making quantitative comparisons of characteristics or similarity [5]. Data clustering is an unsupervised machine learning task, which does not need pre-defined data with labels. A typical cluster analysis pipeline includes feature extraction and selection (aforementioned), proximity measurements, and clustering algorithms. In this case, we choose Euclidean distance to measure the proximity between objects in a vector space. Given two feature vectors *x* and *y*, the Euclidean distance can be calculated as follows:
  (1) $$dist\left(x,y\right):=\sqrt{\sum_{i=1}^{n}{\left(x_{i}-y_{i}\right)}^{2}}.$$
  Complete linkage is one of the most well-known clustering algorithms, which is widely used in biology for structuring and interpreting data in a hierarchical clustering way [6]. This method defines the cluster distance between two clusters to be the maximum distance between their individual components. Given two clusters $C_{1}$ and $C_{2}$, the distance between them can be calculated as follows:
  (2) $$\delta \left(C_{1},C_{2}\right):=\arg\max\left|dist\left(x,y\right)\right|\;x\in C_{1},y\in C_{2},$$
  where |dist(x,y)| denotes the distance between any two objects *x* and *y* in two clusters, respectively.

## 3. Results

**Figure 2:**
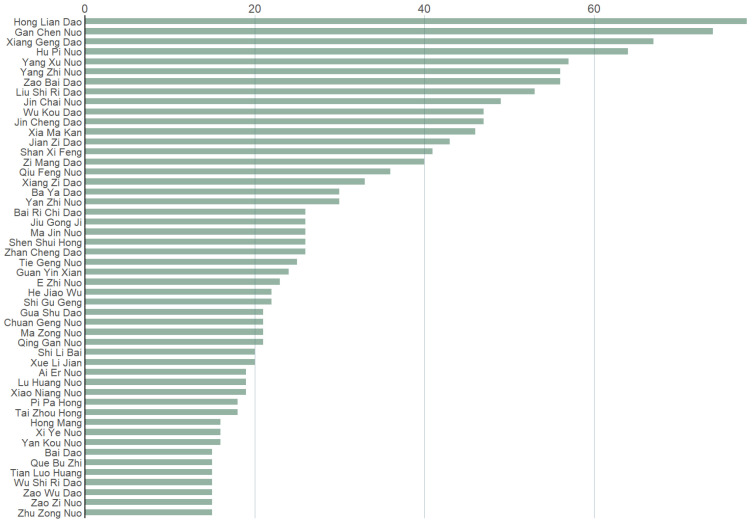
Top 50 rice cultivars with the highest occurrences in our dataset. *x*-axis from top to bottom corresponds to the top 1–50 cultivars in a descending order. *y*-axis corresponds to the number of records of each cultivar in our dataset.

After preprocessing, our dataset currently contains 3773 records of rice cultivars. We count the occurrences of different cultivars, sort them in a descending order, and select the top 50 with the highest frequencies (see Figure 2). It should be noted that many indica cultivars in the dataset were labeled as japonica rice and only the japonica ones with small grains were named “indica”. Concerning this situation, we mark the rice type of both japonica and indica cultivars in the dataset as “japonica/indica”.

In addition, the early-, medium-, and late-maturing rice in the historical gazetteers were differentiated by sowing and harvest seasons. For most early- and medium-maturing rice in local gazetteers, April to May was the sowing time and July to September was the harvesting time. However, for the late-season rice, usually June to July was the sowing time and October to November was the harvesting time. Thus, many cultivars classified as early-season rice in the past are nowadays regarded as medium- or late-maturing cultivars based on their growth duration. Since our focus of this study is rice information in historical texts, we still keep the “pre-modern” concepts of early-/mid-/late- season rice.

**Figure 3:**
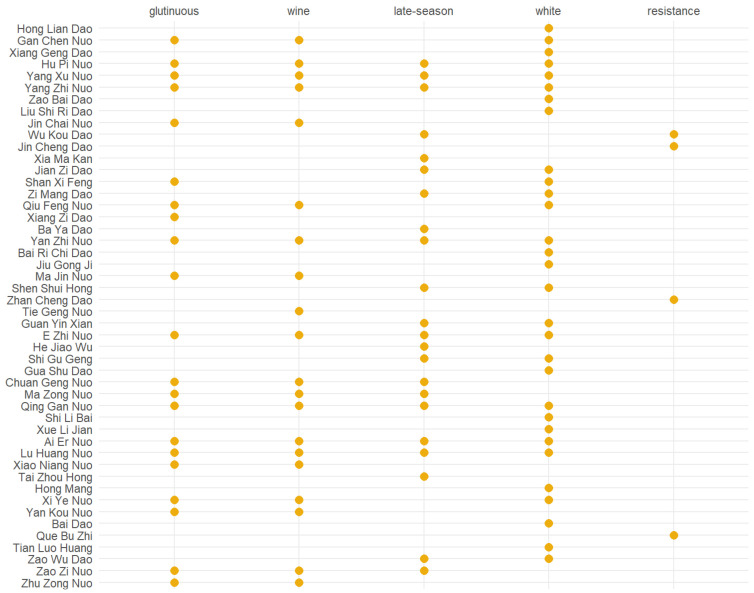
An example of rice features we manually annotate for each cultivar in the dataset. Each yellow dot shows that a certain cultivar contains a specific feature.

Then, we manually annotate ten rice features, such as rice color, type, and growth duration, and use Figure 3 to display five typical characteristics of cultivars in Jiangsu Province. In this dot plot, the *y*-axis represents the rank of top-50 cultivars in our dataset, and each yellow dot shows whether a certain cultivar contains a certain attribute. For example, the top one on y-axis in Figure 3 is a japonica/indica cultivar, which is early-season maturing with a white-rice color.

From Figure 3, we can find that, among the top 50 rice cultivars, white rice appears more often than rice in other colors. In pre-modern China, rice in white color is usually regarded as top-ranking rice and more probably marked as tributes [7]. In addition, the type, uses, and growth duration of rice are frequently mentioned for the top 50 cultivars. We think these features represent the important factors for people to rate different cultivars in historical times.

**Figure 4:**
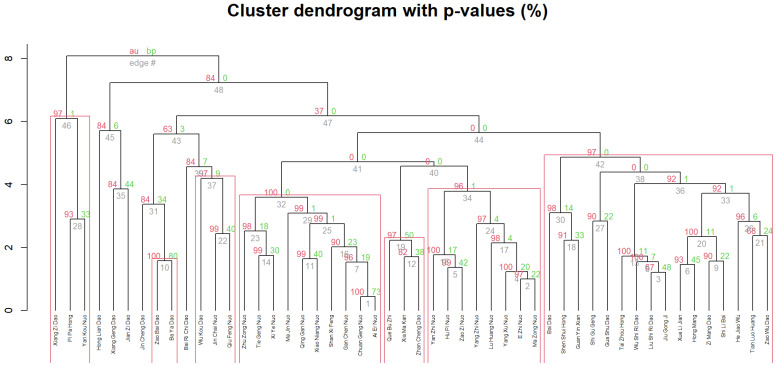
Clustering result in a hierarchical tree structure with marked *p*-value. AU (approximately unbiased) *p*-values are in red, BP (bootstrap probability) values are in green, and cluster labels are in grey. Clusters with AU ≥ 95% are framed by the rectangles.

Furthermore, we use annotated rice features as clustering vectors and four packages in language R are employed to perform hierarchical clustering. Our major steps are listed as follows:

- Before clustering, we use function hopkins() in R package hopkins to calculate the clustering tendency of our dataset. The output is 0.90, which shows that our dataset contains obvious (non-random) cluster structures.
- Then, we use function clValid() in R package clValid to compare different clustering algorithms (e.g., hierarchical clustering, kmeans, and pam) and select the most appropriate one for our experiment. The result shows that hierarchical clustering is the most suitable algorithm for our data.
- Furthermore, function dist() and hclust() in R are used to compute the distance between every pair of cultivars and group them into a hierarchical tree structure based on the corresponding distance information.
- Finally, pvclust function in R package pvclust is used to compute the *p*-value for each cluster, which is visualized in Figure 4.

In Figure 4, *y*-axis of the dendrogram represents the distance of split or merge of clusters. The *x*-axis corresponds to the top 50 rice cultivars in the dataset. This dendrogram re-orders the rows (rice names) of the data matrix, in order to put similar objects close to each other according to the clustering result. As shown in this dendrogram, most clusters framed by rectangles are with *p*-value ≥ 95% (in red), which means most clusters are highly supported by the data.

## 4. Discussion

The compilation of seed catalogs and local gazetteers in China was seriously interrupted between 1966 and 1976. Not only was the work of seed records and gazetteers suspended during that period, but also part of the corresponding archives was destroyed. Although the compilation of records was revived in the late 1970s, the seed catalogs are still not continuous, and the missing records could not be found. Thus, in this paper, we focus on the rice cultivars recorded in local gazetteers of Jiangsu Province from the 12th to 20th century. It is our presumption that two rice cultivars with similar genetic patterns might share similar phenotype information. Although we cannot directly identify the genetic information from historical gazetteers, the semantic similarities between different rice descriptions might help us to discover the potential match between “pre-modern” rice and “present-day” seeds. By extracting, normalizing, and clustering the rice data collection in NJAU, we have two interesting findings, which are discussed as follows.

- **Early/Late-maturing Rice**. In pre-modern China, there was a strong tendency for farmers to plant early-season rice because of the heavy taxes and famine caused by extreme climate events. However, in Figure 3, we notice that the amount of late-maturing rice is more or less the same as early-/mid-maturing rice. In our opinion, when people wrote about rice in the local gazetteers, they paid more attention to the good qualities, high yields, and special characteristics of cultivars than to the “season-type” of rice.
- **Non-Local Rice Cultivars**. In the “top rice” list, we notice that several cultivars on the top-list, such as “Hong Lian Dao”, “Jin Cheng Dao”, and “Zhan Cheng Dao” are not local plants in Jiangsu. For instance, cultivar “Jin Cheng Dao” (see Figure 4), is not a local rice variety in Jiangsu Province but actually came from Guangdong Province [8]. Although Jincheng did not have good textures, it had multiple characteristics, such as strong adaptability to survive in different environments, salt-tolerance, and drought-endurance, which makes it special and might explain why it was mentioned frequently in the gazetteers of Jiangsu.

We use the clustering technique to help us observe the similarities and dissimilarities between cultivars. For instance, in Figure 4, “Liu Shi Ri Dao” and “Jiu Gong Ji” are close to each other since they are both japonica early-season rice. If we match them with extant rice seeds by name and phenotype information, it might not be strictly one-to-one matching, but could have multiple “modern” matching candidates. By calculating the genetic relations between these “modern” seeds, we can see their latent relations, which might in turn help us to further investigate the relations between cultivars in historical records from an evolutionary perspective. Therefore, our next step will involve the participation of biologists, in order to facilitate the knowledge discovery of historical literature with more biological evidence.

## Data Availability

The data presented in this study are available upon request from the corresponding author.
